# Characterization of phenotype markers and neuronotoxic potential of polarised primary microglia *in vitro*

**DOI:** 10.1016/j.bbi.2013.02.005

**Published:** 2013-08

**Authors:** Vibol Chhor, Tifenn Le Charpentier, Sophie Lebon, Marie-Virgine Oré, Idoia Lara Celador, Julien Josserand, Vincent Degos, Etienne Jacotot, Henrik Hagberg, Karin Sävman, Carina Mallard, Pierre Gressens, Bobbi Fleiss

**Affiliations:** a Inserm, U676, Paris, France; b University Paris Diderot, Sorbonne Paris Cité, UMRS 676, Paris, France; c PremUP, Paris, France; d Perinatal Centre, Institute of Neuroscience and Physiology and Clinical Sciences, Sahlgrenska Academy, University of Gothenburg, Gothenburg, Sweden; e Department of Reproductive Biology, Imperial College London, United Kingdom; f Centre for the Developing Brain, Department of Perinatal Imaging and Health, King’s College London, United Kingdom

**Keywords:** Interleukins, Inflammation, Lipopolysaccharide, M1–M2, Macrophage, Neuroinflammation, Neuroprotection, Neuronal cell death, TLR4, Drug-screening

## Abstract

Microglia mediate multiple facets of neuroinflammation, including cytotoxicity, repair, regeneration, and immunosuppression due to their ability to acquire diverse activation states, or phenotypes. Modulation of microglial phenotype is an appealing neurotherapeutic strategy but a comprehensive study of classical and more novel microglial phenotypic markers *in vitro* is lacking. The aim of this study was to outline the temporal expression of a battery of phenotype markers from polarised microglia to generate an *in vitro* tool for screening the immunomodulatory potential of novel compounds. We characterised expression of thirty-one macrophage/microglial phenotype markers in primary microglia over time (4, 12, 36, and 72 h), using RT-qPCR or multiplex protein assay. Firstly, we selected Interleukin-4 (IL-4) and lipopolysaccharide (LPS) as the strongest M1–M2 polarising stimuli, from six stimuli tested. At each time point, markers useful to identify that microglia were M1 included iNOS, Cox-2 and IL-6 and a loss of M2a markers. Markers useful for quantifying M2b-immunomodulatory microglia included, increased IL-1RA and SOCS3 and for M2a-repair and regeneration, included increased arginase-1, and a loss of the M1 and M2b markers were discriminatory. Additional markers were regulated at fewer time points, but are still likely important to monitor when assessing the immunomodulatory potential of novel therapies. Further, to facilitate identification of how novel immunomodulatory treatments alter the functional affects of microglia, we characterised how the soluble products from polarised microglia affected the type and rate of neuronal death; M1/2b induced increasing and M2a-induced decreasing neuronal loss. We also assessed any effects of prior activation state, to provide a way to identify how a novel compound may alter phenotype depending on the stage of injury/insult progression. We identified generally that a prior M1/2b reduced the ability of microglia to switch to M2a. Altogether, we have characterised a profile of phenotype markers and a mechanism of assessing functional outcome that we can use as a reference guide for first-line screening of novel immunomodulatory therapies *in vitro* in the search for viable neuroprotectants.

## Highlight

► A unique catalogue of phenotype markers and neuronotoxic effects of polarised primary microglia, as a comparative tool to screen neurotherapies.

## 1 Introduction

In response to insult or injury microglia and macrophages are capable of acquiring diverse and complex phenotypes, allowing them to participate in the cytotoxic response, immune regulation, and injury resolution. Nomenclature of these phenotypes varies across the literature (Kreider et al., 2007; Martinez et al., 2008) but can be characterised into four main states, classically activated M1 with cytotoxic properties; M2a with an alternate activation and involved in repair and regeneration; M2b with an immunoregulatory phenotype; or M2c with an acquired-deactivating phenotype. The concept of phenotype, including nomenclature, and its importance in our understanding of injury processes and drug design and has been extensively reviewed elsewhere (Mosser and Edwards, 2008; Martinez et al., 2009; Ransohoff and Perry, 2009; Perry et al., 2010; Weinstein et al., 2010).

Microglia are self-renewing and long-lived resident macrophage-like cells of the brain (Giulian and Baker, 1986; Glenn et al., 1992; Kettenmann et al., 2011). In addition to important roles in inflammation, microglia are also critical in developmental processes such as synaptogenesis, and are imperative for the maintenance of neural homeostasis (see, Tremblay et al., 2011; Ekdahl, 2012). Importantly, microglial phenotypes reflect expression of cell surface receptors and release of soluble factors with recognised functions. Over time following injury and over time *in vitro* in response to stimuli, expression of the markers used to characterise these states changes (Perego et al., 2011; Hu et al., 2012). Beneficial for our ability to study these populations, phenotypes have known prototypical inducers; Toll-like-receptor-4 agonist lipopolysaccharide (LPS), IFNγ and TNFα for M1; IL-4 and IL-13 for M2a; immune complexes and toll-like-receptor agonists, and IL-1R ligands for M2b; and IL-10, TGF-β and glucocorticoids for M2c. However, a great deal of the studies outlining these inducers were performed in peritoneal or blood derived macrophages, and microglia are known to differ in their responsiveness to stimuli (Schmid et al., 2009).

Inflammatory processes, including activation of microglia, are initiated in the adult and perinatal brain after most (if not all) types of insults and injury (see, Degos et al., 2010; Perry et al., 2010; Bilbo et al., 2012; Fleiss and Gressens, 2012; Hagberg et al., 2012). The importance of microglia in protecting the brain is illustrated by the observation that complete blockade of microglial activity exacerbates brain damage in adult and neonatal hypoxic ischemic injury models (Lalancette-Hebert et al., 2007; Faustino et al., 2011). Also, it is possible for neurotherapeutics to be effective in the neonatal brain without reducing microglial number, also suggesting that modulation rather than suppression may be important (Doverhag et al., 2010; Fleiss et al., 2012). In fact, cell therapy with microglia has been shown to reduce injury in a model of adult hypoxic/ischemic injury (Imai et al., 2007). All together these observations support the need for screening tools to identify neurotherapeutics that support a repair and regenerative microglial phenotype.

Cultured primary microglia are a useful first line research tool with which to screen the immunomodulatory potential of novel therapeutics. Primary microglial cultures derived from early postnatal rodents have cell surface receptor expression and functional characteristics similar to that seen in microglia *in vivo* (Giulian and Baker, 1986; Henn et al., 2009). However, despite the enormous wealth of information regarding macrophage/microglial phenotype, to the best of our knowledge there is no comprehensive time and stimulus dependent data on the phenotype of primary microglia. In addition, a great deal of our understanding of phenotype is derived from studies of macrophages. However, the responses of resident microglia differ from that of the circulating macrophages that infiltrate the brain during insult/injury (Lalancette-Hebert et al., 2007; Schmid et al., 2009), suggested to relate to differing origin of these cell types (Saijo and Glass, 2011). As such, it is critical to understand the specific functions and responses of microglia. Thus, the aim of this study was to characterise in primary microglia the expression of a battery of classic (IL-6, iNOS etc.) and more novel phenotype markers (SphK1/2, FIZZ1 etc.) in a time and stimulus dependent manner and to create a functional output with which to evaluate the neuronotoxic effects of phenotype. We propose that this data set represents an *in vitro* model, useful as a first-line screening tool, to assess the immunomodulatory potential of novel neurotherapeutics in microglia of an M1 or M2 phenotype.

## 2 Methods

### 2.1 Animals

Animals were handled according to institutional guidelines of Institut National de la Santé et de la Recherche Scientifique (Inserm) France, or the Gothenburg University Sweden Animal Ethics Committee and met the guidelines for the Care and use of laboratory animals (NIH, Bethesda, Maryland, USA). Experiments were performed using OF1 strain mice from Charles River (L’Arbresle, France).

### 2.2 Drugs

Lipopolysaccharide (LPS; Sigma, Lyon, France, L2880, lot 050M4014) was diluted in PBS to a stock concentration of 0.1 mg/mL. Cytokines were from R&D systems (Lille Cedex, France), and diluted in PBS and 0.1% bovine serum albumin to create stock solutions; 5 μg/mL for IL-1β, 1 μg/mL for tumour necrosis factor-α (TNFα) and 2 μg/mL for IL-4; IL-10; and Interferon-γ (IFNγ).

### 2.3 Primary microglial culture

Primary mixed glial cell cultures were prepared from the cortices of postnatal day (P) 0–1 OF1 mice, as previously described (Thery et al., 1991). Pups of both sexes were included and on average an equal number of males and females were included in each culture. After dissection of the cortices in 0.1 M PBS with 6% glucose and 2% penicillin–streptomycin (PS; Gibco, Cergy Pontoise, France) and removal of the meninges, the cortices were chopped into small pieces and subsequently mechanically dissociated. The suspension was diluted in pre-cooled low glucose Dulbecco’s modified Eagle’s minimum essential medium (DMEM, 31885, Gibco) supplemented with 10% foetal bovine serum (FBS, Gibco) and 0.01% PS. Microglia were isolated from primary mixed glial cultures on day *in vitro* 14 (DIV14) using a reciprocating shaker (20 min at room temperature) and repeated rinsing with their medium using a 10 mL pipette. Media was subsequently removed, microglia pelleted via centrifugation (300*g* × 10 min) and following resuspension maintained in DMEM supplemented with 10% FBS at a concentration of 4 × 10^5^ cells/mL in 6-well culture plates. Culture purity was verified by immunostaining (*n* = 5) using cell-type specific antibodies against tomato lectin (microglia), glial fibrillary acidic protein (GFAP; astrocytes) and neuronal nuclear antigen (NeuN; neurons) and revealed a >99% purity of microglia.

**Fig. 1:**
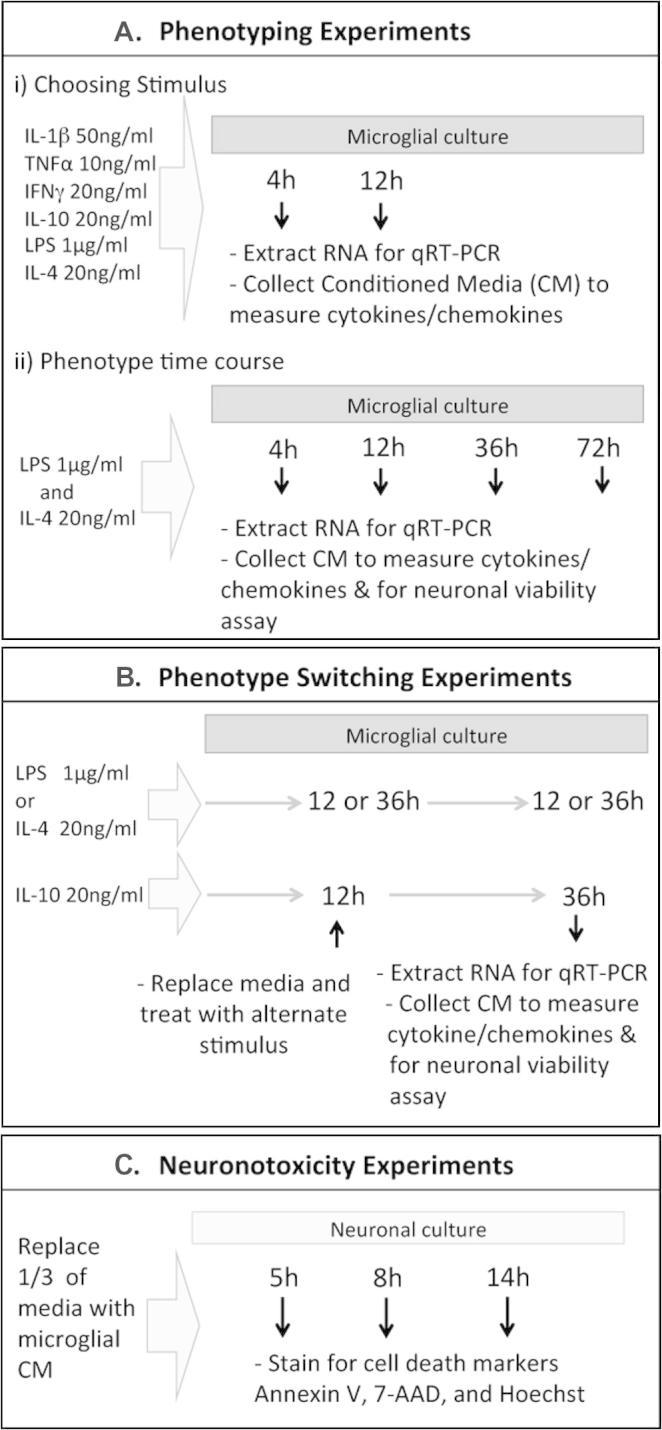
Schematic representation of the experimental setups, including phenotyping, neuronal viability and phenotyping switching experiments.

Based on previous reports, the day after plating, microglia were treated with PBS (vehicle; 10 μl/ml of culture media), LPS at 1 μg/mL (Takeuchi et al., 2006; Kaindl et al., 2012), IL-1β at 50 ng/mL (Street et al., 2003; Hjorth et al., 2010), IL-4 at 20 ng/mL (Butovsky et al., 2006; Kigerl et al., 2009), IL-10 at 20 ng/mL (Strle et al., 2002), TNFα at 10 ng/mL (Bernardino et al., 2005), or IFNγ at 20 ng/mL (Butovsky et al., 2006; Kigerl et al., 2009). After various exposure times (4, 12, 36, and 72 h) supernatant (conditioned media) was collected and stored at −80 °C until analysis of cytokine/chemokine levels or for use in neuronal viability studies, and cells were harvested and RNA extracted for gene expression analysis. A schematic representation of the experimental timelines is shown in Fig. 1.

### 2.4 Primary neuronal cultures

Cultured neurons were derived from the cerebral cortex of embryonic (E) 14.5 mice as previously described (Fontaine et al., 2008). After dissection of the cortices and removal of the meninges the cortices were minced into small pieces, chemically dissociated with 0.25% trypsin (Gibco) and 1% DNase (Gibco) at 37 °C for 20 min. The reaction was stopped by addition of 0.001% horse serum, and cortex pieces were subsequently mechanically dissociated. Cells were seeded in 8-well Ibitreat slide (Ibidi®, Biovalley, Marne-La-Vallée, France) pre-coated with 30 μg/mL poly-DL-ornithine (PO, Sigma) at 12.5 × 10^4^ cells per well to a final volume of 250 μL per well. Cells were cultured in Neurobasal Medium (Gibco) supplemented with 2% B27 (Gibco), 1% glutamine 100× (Gibco) and 0.05% PS. One third of the culture medium was exchanged for fresh solution twice a week and arabinocytidine hydrochloride (Ara C: 5 μM, Sigma, C1768) was added at DIV4.

At DIV6, one third of neuronal media was exchanged with conditioned media from microglia exposed to LPS for 12 h or IL-4 for 36 h, as described above. At 5, 8, and 14 h after exposure to conditioned media neurons were stained with markers of cell death, using the protocol described below.

### 2.5 RNA extraction and quantification of gene expression by real-time qPCR

Total RNA from primary microglial cell cultures was extracted with the RNeasy mini kit according to the manufacturer’s instructions (Qiagen, Courtaboeuf, France). RNA quality and concentration were assessed by spectrophotometry with the Nanodrop™ apparatus (Thermoscientific, Wilmington, DE, USA). Total RNA (1–2 μg) was subjected to reverse transcription using the iScript™ cDNA synthesis kit (Bio-Rad, Marnes-la-Coquette, France). RT-qPCR was performed in duplicate for each sample using SYBR Green Supermix (Bio-Rad) for 40 cycles with a 2-step program (5 s of denaturation at 96 °C and 10 s of annealing at 60 °C). Amplification specificity was assessed with a melting curve analysis. Primers were designed using Primer3 software, and sequences and their NCBI references are given in Supplementary Table 1. The relative expression of genes of interest (GOI) were expressed relative to expression of the reference gene, Glyceraldehyde 3-phosphate dehydrogenase (*GAPDH*). Analyses were performed with the Biorad CFX manager 2.1 software.

### 2.6 Multiplex cytokine/chemokine assay

Microglia media harvested at different time-points following treatment initiation was centrifuged briefly to remove particulates (300*g* for 10 min). Cytokine and chemokine levels in the microglial supernatant were measured using a Bio-plex 200 with a 96-well magnetic plate assay according to the manufacturers instructions (Biorad laboratories, Marnes la Coquette, France). Cytokine and chemokine measured included IL-1α, IL-1β, IL-2, IL-6, IL-10, IL-12 (p70), IL-13, G-CSF, GM-CSF, IFNγ, TNFα, CXCL1 (KC), CCL2 (MCP-1), and CCL5 (RANTES). All samples were run in duplicates and data was analysed with the Bio-Plex Manager software.

### 2.7 Cell viability (mitochondrial activity) assay

Microglial viability was quantified using the colorimetric CellTiter 96® A Queous Non-Radioactive Cell Proliferation Assay (Promega, Madison, WI, USA) per manufacturer instructions. In this assay, MTS a tetrazolium dye is bioreduced by the mitochondria into a formazan product that is soluble in tissue culture medium. In brief, 20 μL of the MTS solution was added to each well of a 96-well-plate containing 40 × 10^3^ microglial cells 12 h following treatment with PBS, LPS or IL-4 and the absorbance of formazan was measured 1 h later at 490 nm using the Beckman Coulter Paradigm Detection Platform (Beckman, Fullerton, CA, USA).

### 2.8 Fluorescence immunohistochemistry for cell death markers in neurons

Neurons were stained at various time points following exposure to microglial conditioned media, to assess the type and percentage of neuronal cell death. Loss of plasma membrane integrity was detected through increased permeability to 7-Amino Actinomycin D (7-AAD, 1 μg/mL; Molecular Probes, A1310). Externalisation of phosphatidylserine (PI; Molecular Probes, A13201), a characteristic of apoptosis, was detected with Alexa Fluor 594-conjugated-Annexin V (1/60 dilution; Molecular Probes, A13201). Nuclear morphology and total cell number was visualised using the DNA binding dyes Hoechst 33342 (1 μg/mL; Sigma, B2261). Neurons were cultured at 37 °C for 30 min with 7-AAD, Annexin V and Hoechst and following thorough washing with PBS, fluorescence microscopy was performed with a Nikon Eclipse Ti-TIRF at a 20× objective. 7-AAD was observed using 528–553 nm excitation/590–650 nm emission filter, Annexin V at 465–495 nm excitation/515–555 nm emission filter and Hoechst at 340–380 nm excitation/435–485 nm emission filter. Images for analysis were acquired with a Coolsnap HQ2 camera and staining assessed with the Nikon NIS Elements and Image J software. Neurons were classified into one of four populations; (i) cells negative for 7-AAD and Annexin V (viable cells), (ii) cells positive only for Annexin V (early apoptotic), (iii) cells positive to both Annexin V and 7-AAD (late apoptotic or necrotic), and, (iv) cells positive only to 7-AAD (early necrotic). Data for each marker was adjusted to the total number of Hoechst positive cells per field of view.

### 2.9 Fluorescence immunohistochemistry of microglia

To immunolabel microglia, cell medium was removed and cells were fixed with Histofix (Histolab, Sweden, Gothenburg). Fixed cells were treated with PBS-Triton X 100 (0.5%) for 20 min and primary antibodies applied overnight at 4 °C. Antibodies used included; Monoclonal Mouse anti-Arginase 1, 1:50, 610708, (BD Biosciences, London, UK); Monoclonal Rat anti-CD86, 1:100, 550542, (BD Biosciences); Polyclonal Rabbit anti-iNOS, 1:50, 610332, (BD Biosciences); Polyclonal Goat anti-IL-1RA, 1:400, Sc-8482, (Santa Cruz, CA, USA); Polyclonal Rabbit anti-Cox-2, 1:400, Ab15191, (Abcam, Cambridge, UK), and; Polyclonal Rabbit anti-Ki67, NCL-Ki67-P, 1:1000, (Novocastra, Newcastle Upon Tyne, UK). After thorough washing the appropriate Alexa 488 or 594 conjugated fluorescent secondary antibody (1:500, Invitrogen, Stockholm, Sweden) was applied for 1 h at room temperature. Specificity of staining was checked by including no-primary and no-secondary antibody controls during staining. Cells were coverslipped with Prolong Gold DAPI (Invitrogen) or Hoechst, and analysed using Zeiss Axio Imager.Z2 with the ZEN 2011 Blue software.

### 2.10 Statistics

Data are from three or more independent microglial or neuronal cultures and are presented as mean ± SEM. Data were assessed for normality. Gene and protein expression over time was analysed with an ANOVA and a Bonferroni post-test. Neuronotoxicity was analysed with a Kruskal–Wallis and a Dunns post-test. Switching effects on phenotype was analysed via a Student’s *t*-test or Mann–Whitney test. Significance from the Student’s *t*-test, Mann Whitney or the ANOVA post-test is shown on the graphs (^§^*p* = 0.05; ^∗^*p* < 0.05; ^∗∗^*p* < 0.01; ^∗∗∗^*p* < 0.001). Statistics used for each data set are indicated in the figure legends. Analyses were performed with Graphpad 5.0 software (San Diego, CA, USA), and *p* ⩽ 0.05 was accepted as statistically significant.

### 2.11 Nomenclature of microglial phenotype

We have adopted nomenclature consistent with the separate works of Mantovani, Gordon, Colton, and Perry (Mantovani et al., 2004; Colton, 2009; Martinez et al., 2009; Ransohoff and Perry, 2009); classically activated M1; M2a alternate activation repair/regeneration; M2b immunoregulatory; and M2c acquired-deactivating. However, due to a great deal of overlap between the phenotypic markers for M2b and M2c we have chosen to discuss M2c only in the context of IL-10 dependent effects, but not ascribe particular markers to this phenotype.

## 3 Results

### 3.1 LPS and IL-4 induce robust M1/2b and M2a phenotypes respectively

Our first aim was to determine which stimuli from a selection of those previously used to polarise microglia/macrophages would most clearly polarise microglia in our culture conditions (i.e., to a cytotoxic M1 vs. repair and deactivating M2 phenotype). We exposed primary microglia to LPS, IL-1β, TNFα, IL-10, IL-4 or IFNγ for either 4 or 12 h and assessed gene expression of 17 phenotype markers and protein levels of 7 phenotype markers, the proposed functions of which are outlined in Table 1. Markers were characterised as M1, M2a or M2b based on previous reports (Mantovani et al., 2004; Martinez et al., 2009; Ransohoff and Perry, 2009; Colton and Wilcock, 2010). Patterns of gene and protein expression were similar at 4 and 12 h (Table 2; Supplementary Table 2).

Prototypical pro-inflammatory stimuli (LPS, IL-1β and TNFα), increased gene expression of known cytotoxic M1 markers, Cox-2 and iNOS and as well as release of CXCL1/KC, TNFα, IL-6, and IL-1β proteins (Table 2). Moreover, exposure to LPS, IL-1β, and TNFα increased expression of immunomodulatory M2b markers, (IL-1Rn and SOCS3) and caused a decrease in the expression of M2a-repair/regeneration markers. The magnitude of these increases in M1 and M2b markers was greatest in LPS stimulated microglia (Table 2).

**Fig. 2:**
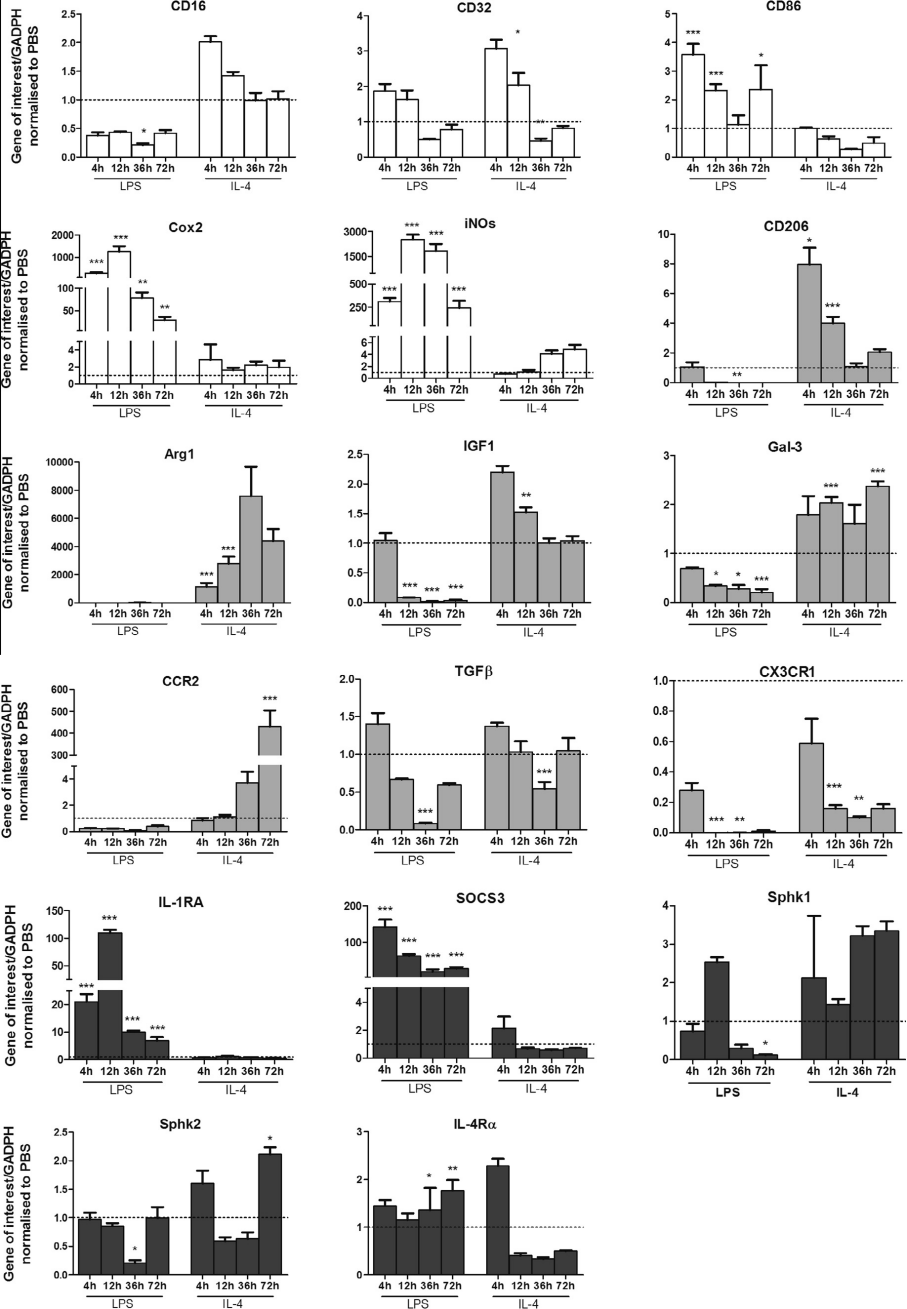
Gene expression of phenotype markers over time in response to M1 or M2 inducing conditions. Expression of phenotype markers grouped as M1 (White), M2a (grey) or M2b (black) dependent on their proposed function, in primary microglia exposed to M1/2b inducing conditions (+LPS) or M2a inducing conditions (+IL-4), for 4 h, 12 h, 36 h or 72 h. Expression shown relative to PBS only, dotted line. Data are mean ± SEM of at least 3 experiments. Data were assessed via an ANOVA, and where significant the results of the Bonferroni post-test are shown; ^∗^*p* < 0.05; ^∗∗^*p* < 0.01; ^∗∗∗^*p* < 0.001, compared to PBS.

IL-10 was the only stimuli to induce gene expression for IL-4Rα at 12 h (Table 2), and of note expression was still greater than 3-fold above control at 36 h (data not shown). The response to IL-10 was also unique in that it was the only stimuli to increase gene expression for markers ascribed to M2b immunomodulatory markers at 12 h, but did not increase either cytotoxic M1 or repair M2a markers (Fig. 2E). However, IL-10 induced a similar profile of cytokine/chemokines to that of LPS, TNFα, and IL-1β, (increased IL-6, CXCL1, and CCL2) although the levels were considerably lower. (Table 2; Supplementary Table 2).

The known anti-inflammatory cytokine IL-4 was the only stimulus that increased gene expression of M2a-repair/regeneration phenotype markers; arginase-1 (Arg1) and CD206. Also, IL-4 was the only stimulus that did not increase gene expression for M2b-immunomodulatory phenotype markers (Table 2; Supplementary Table 2). IL-4 did not increase release of any cytotoxic M1 markers, such as IL-1β, IL-6, TNFα or CXCL1 (Table 2; Supplementary Table 2).

Collectively from this data we concluded that each of the pro-inflammatory stimuli (LPS, IL-1β, TNFα or IFNγ) induced markers of both cytotoxic M1 and immunomodulatory M2b activation state. However, the magnitude of marker increase was highest for LPS and this was chosen as our pro-inflammatory stimulus for later experiments. IL-4 was designated our anti-inflammatory inducing stimulus due to its specific actions to increase markers known to indicate functions of repair and regeneration.

### 3.2 Gene expression levels discriminate between microglial phenotypes

Using our selected M1/2b and M2a inducing stimuli (+LPS and +IL-4, respectively) we investigated the temporal profile of expression of phenotype markers (at 4, 12, 36, and 72 h), via gene expression (Fig. 2). We sought to determine how many of the classical and more novel markers would have predictive value in this *in vitro* system for characterising phenotype.

**Supplementary Fig. 1:**
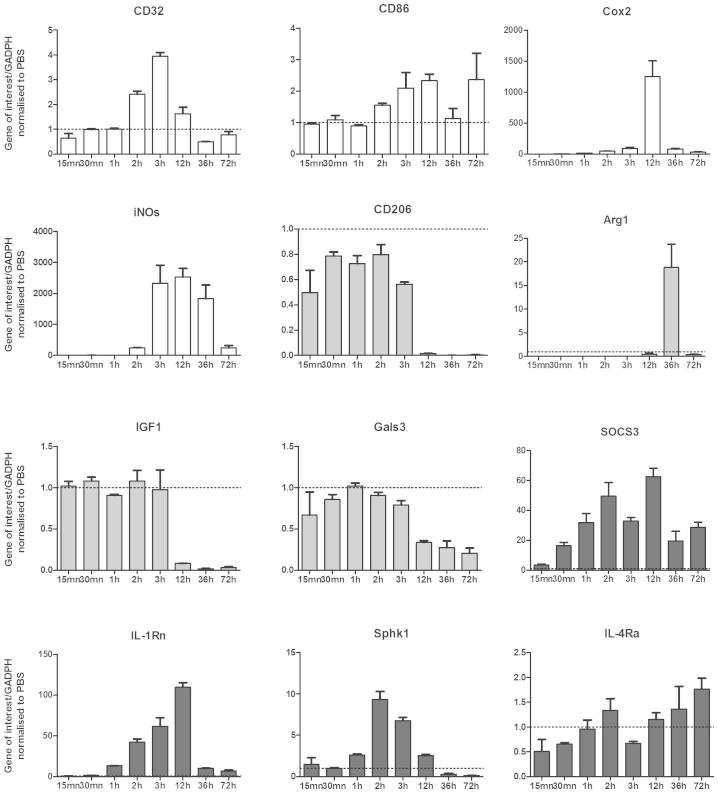
In response to LPS gene expression for M1 and M2b are induced within 2 h of stimulation. Gene expression of markers categorized as M1 (white bars), M2a (grey bars) and M2b (black bars) in primary microglia exposed to M1/2b inducing condition (+LPS). Expression shown relative to PBS only, dotted line. Data are mean ± SEM of at least 3 independent experiments. Data were assessed via an ANOVA, and where significant the results of a Bonferroni post-test are shown; ^∗^*p* < 0.05; ^∗∗^*p* < 0.01; ^∗∗∗^*p* < 0.001, compared to PBS.

We identified several key strong discriminatory markers for phenotype over time. In response to the prototypical microglial activator LPS, gene expression of the M1 markers iNOS, CD86 and Cox-2 were increased at 4 h, and iNOS and Cox-2 remained elevated at each subsequent time point assessed. Similarly, the M2b markers IL-1RA and SOCS3 were also elevated at 4 h and remained elevated following exposure to LPS. To ensure that the apparent co-incident induction of M1 and M2b was not due to the first sample being at 4 h after stimulation, samples were taken from 15, 30, 60, 120, and 240 min after stimulation. This revealed that markers of both phenotypes were induced in parallel, and within 120 min (Supplementary Fig. 1). Overall, in response to the M1/2b inducer LPS, gene expression for M2a markers were strongly reduced over time (Fig. 2).

**Supplementary Fig. 2:**
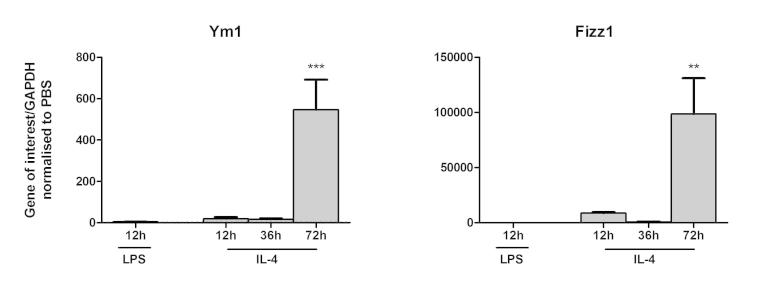
Expression of YM1 and Fizz1 are increased after 72 h of M2a inducing conditions. Gene expression for YM1 and Fizz1 in primary microglia stimulated for 12, 36, or 72 hr with either LPS or IL4. Data are mean ± SEM of at least 3 independent experiments assessed via a Kruskal Wallis, and where significant the results of a Dunn’s post test are indicated; ^∗^*p* < 0.05; ^∗∗^*p* < 0.01; ^∗∗∗^*p* < 0.001 compared to averaged PBS in post-hoc.

In M2a inducing conditions (+IL-4), expression of CD206 and IGF-1 was greatest at 4 h and declined over time. However, Arg1 and CCR2 increased with time and were highest at 36 and at 72 h, respectively (Fig. 2). Also, expression was greatly induced at 72 h for the M2a markers FIZZ1 and YM1, but we were unable to find either expressed in basal conditions or after IL-4, with which to normalise expression and they are shown in Supplementary Data (Supplementary Fig. 2). Overall, in response to the M2a inducer IL-4, gene expression for M1/2b markers was stable or reduced.

### 3.3 Classic pro-inflammatory cytokines discriminate between microglial phenotypes

**Fig. 3:**
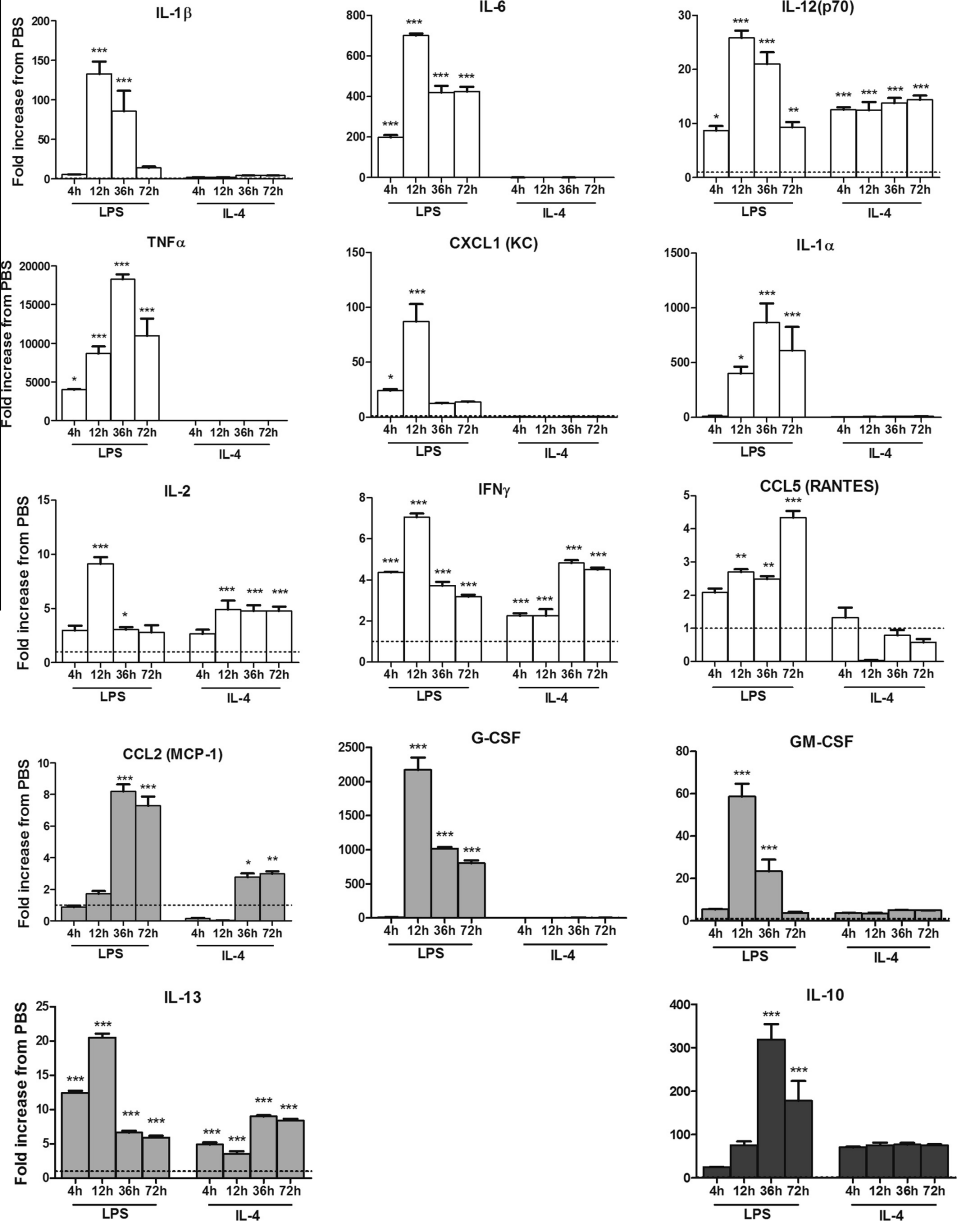
Protein expression for phenotype markers over time in response to M1 or M2 inducing conditions. Expression of phenotype markers in the conditioned media, grouped as M1 (White), M2a (grey) or M2b (black) dependent on their proposed function, from primary microglia exposed to M1/2b inducing conditions (+LPS) or M2b inducing conditions (+IL-4), for 4 h, 12 h, 36 h or 72 h. Expression shown relative to PBS only, dotted line. Data are mean ± SEM of at least 3 experiments. Data were assessed via an ANOVA, and where significant the results of a Bonferroni post-test are shown; ^∗^*p* < 0.05; ^∗∗^*p* < 0.01; ^∗∗∗^*p* < 0.001, compared to PBS.

Expression over time of a number of cytokines/chemokines from stimulated microglia (Fig. 3) had a profile specific to an M1 or M2 phenotype. Exposure to the M1/2b inducer LPS increased expression of all cytokines and chemokines in all phenotype categories, at 12, 36, and 72 h (Fig. 3). IL-4 also increased expression of many of the M1, M2a, and M2b cytokines and chemokines at multiple time points. However, in response to IL-4, the M1 associated factors IL-6, TNFα, and CCL5 were not increased at any assessed time point. As these markers were increased at all time points by LPS they are useful discriminatory markers. The M1 and M2b markers G-CSF, GM-CSF, IL-1β, and IL-1α were also not increased by IL-4, and induced by LPS at 12, 36, and 72 h, proving useful to discriminate between phenotypes at later than 4 h of stimulation.

In summary of the timing of marker expression (measured as either gene or protein expression; Figs. 2 and 3); the majority of M1 markers (10/13) and M2b markers (8/13) were maximally expressed at 12 h of exposure to LPS. In response to IL-4, seven of the twelve M2a markers were increased at 36 h and there was a maximal reduction of the M1/2b markers, CD16, CD32, CD86, IL-4Rα, SOCS3, SphK2. As such, during additional experiments microglia were exposed to 12 h of LPS to induce maximal M1/2b and IL-4 for 36 h for maximal M2a phenotype.

### 3.4 Immunohistochemistry correlated with gene expression of the selected markers

**Supplementary Fig. 3:**
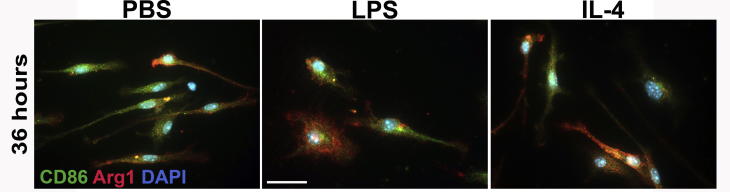
Expression of CD86 did not vary dramatically, and Arg1 increased in microglia exposed to IL4. Microglia were stimulated for 36 h with LPS, IL-4 or with PBS control. CD86 staining in green and Arg1 in red. Photomicrographs taken at 63×, scale bar 25 μm.

**Fig. 4:**
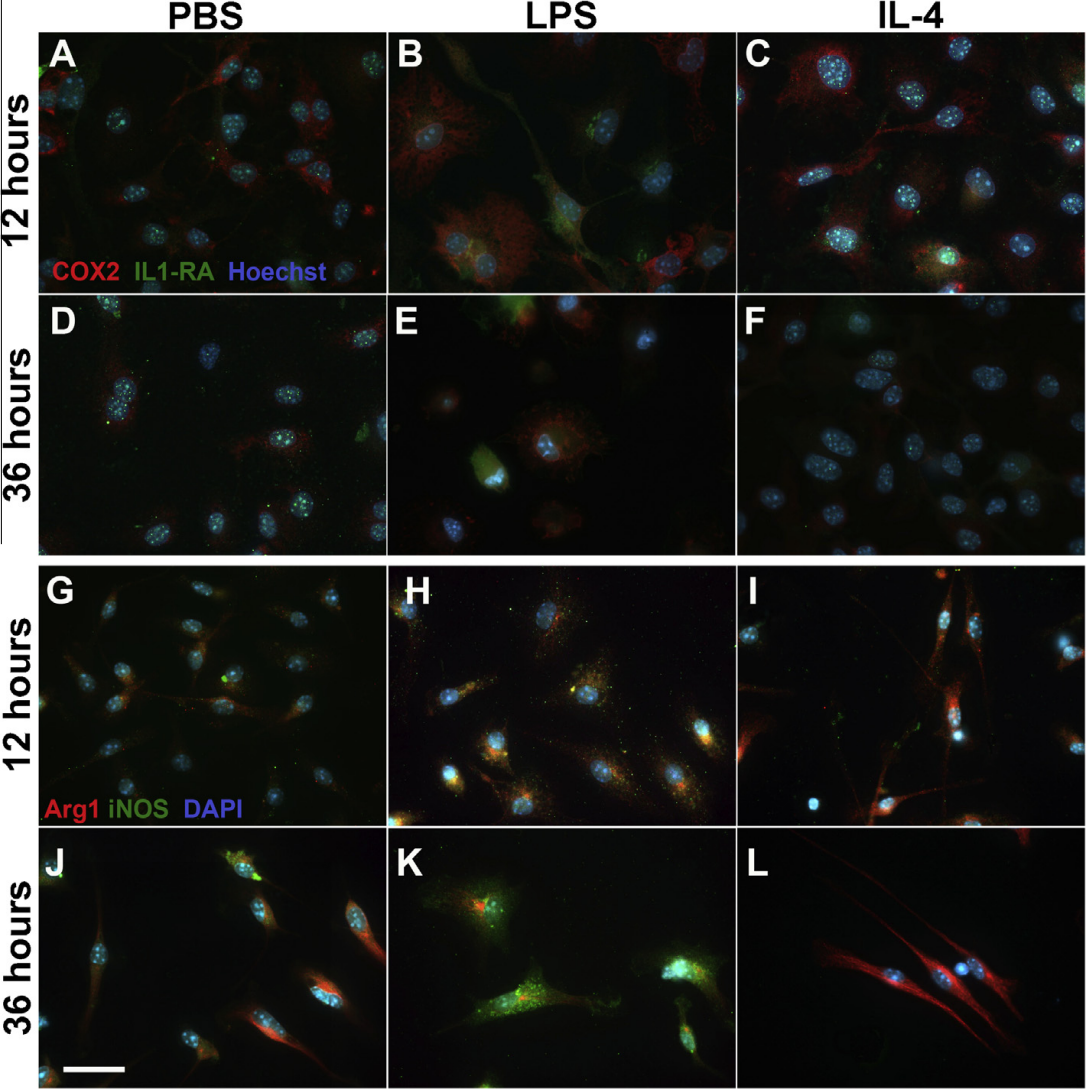
Expression of selected phenotype markers in cultured microglia. Microglia were stained with markers as indicated after exposure for 12 or 36 h to LPS or IL-4, with PBS as control. All photomicrograph were taken at 63× magnification, scale bar 25 μm. Panels A–F, Cox-2 in red, IL-1RA in green and Hoechst in blue. Panels G–L, Arg1 in red, iNOS in green and DAPI in blue.

To identify the distribution of phenotype markers within cells and between cells exposed to LPS or IL-4, microglia were double labelled. Microglia were labelled with an M1 and an M2a or M2b marker i.e., iNOS and Arg1 (Fig. 4A–F), Cox-2 and IL-1RA (Fig. 4G–L) or CD86 and Arg1 (Supplementary Fig. 3).

Cox-2 and IL-1RA expression increased following exposure to LPS, but of interest, it appeared that cells preferentially increased only one of these markers. In some cases microglia appeared to have low levels of IL-1RA and Cox-2 (Fig. 4E). Activation with IL-4 caused IL-1RA to be diffusely distributed within the cytoplasm, whereas in PBS and LPS (at 12 h) treated microglia it appeared as distinct granules near the nucleus. Exposure to IL-4 caused an almost complete loss of IL-1RA and Cox-2 expression at 36 h (Fig. 4D vs. 4F). Nevertheless, at 12 h, compared to PBS it appears there was an induction of Cox-2.

Activation of microglia with IL-4 induced Arg1 and this up-regulation was greatest at 36 h. Activation with LPS or IL-4 caused Arg1 staining to appear as distinct fibres wrapping around the nucleus and extending into the processes, reminiscent of microtubule staining (Fig. 4K and L). For Arg1 expression was reduced in LPS treated cells at 36 h, but surprisingly a very occasional microglia displaying strong staining Arg1 was seen.

Following 12 h of exposure to LPS, expression of iNOS was obviously increased, with the appearance of punctate cytoplasmic granules. At 36 h of LPS, the cells were very intensely stained for iNOS with even larger granules within the cytoplasm surrounding the nucleus, but not into the processes. In contrast, exposure to IL-4 caused an almost complete loss of iNOS at both time points. CD86 expression, as expected from gene data, did not appear to change substantially over time (Supplementary Fig. 3).

**Supplementary Fig. 4:**
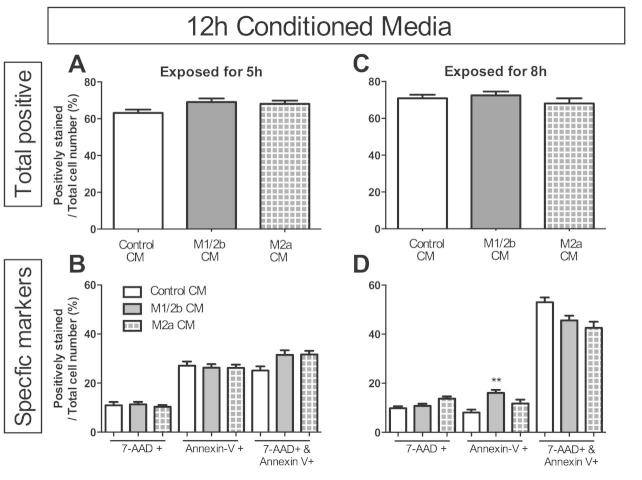
Proliferation was altered when microglial phenotype was polarized, LPS inducing and IL-4 reducing Ki67 expression. Microglia were stained with Ki67 (red) and Hoechst (blue) after exposure for 12 or 36 h to LPS or IL-4, with PBS as control. Ki67 positive cell numbers were counted and expressed as a proportion of total cell number (Hoechst positive). Photomicrograph were taken at 60× magnification, scale bar 100 μm.

In addition, we investigated whether LPS or IL-4 altered expression of Ki67, a marker of proliferation. We observed a significant 25% increase in the number of cells expressing Ki67 at a maximal induction of M1/2b (LPS, 12 h), and a significant 75% reduction where microglia were maximally M2a (IL-4, 36 h) (Supplementary Fig. 4).

### 3.5 Neuronal viability is increased by soluble factors from M2a microglia and decreased by soluble factors from M1/2b microglia

**Supplementary Fig. 5:**
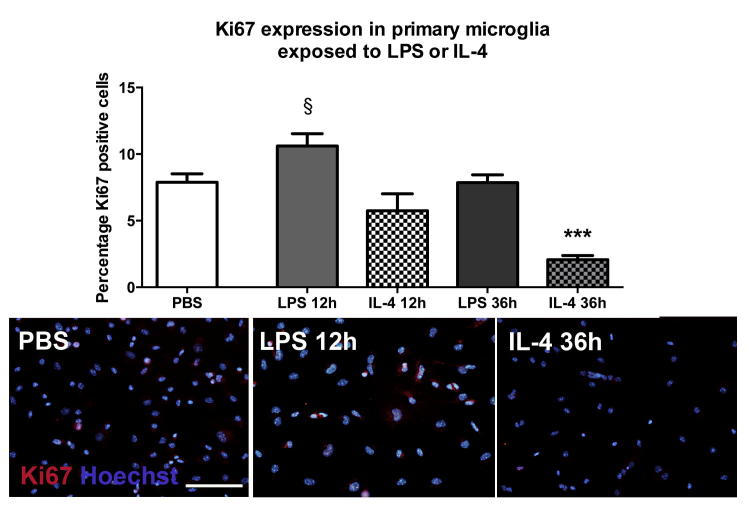
Cell death was largely unaffected by exposure to soluble factors from M1/2a or M2b microglia for 5 or 8 h. Total appearance of cell death markers (top panels) and relative numbers of specific cell death type markers (middle panels) in neurons exposed to conditioned media harvested following 12 h induction to an M1/2b or M2a phenotype, or exposure to PBS. Neuronal viability was assessed after exposure of neurons for 5 h (A, B) or exposure for 8 h (C, D) to the conditioned media. Viability was assessed dependent on positive staining for 7-ADD (necrotic-like death marker), Annexin V (apoptotic-like death marker), or both (late stage cell death indicated).

Neurons were exposed to soluble factors (i.e. conditioned media), from microglia induced to an M1/2b or M2a phenotype with LPS or IL-4, (or PBS as control), in a model of growth factor deprivation mediated neuronotoxicity, as previously described (Dean et al., 2010). In brief, microglia media is deficient in neuronal growth factor supplements and neuronotoxicity was approximately 60% at 24 h in response to switching to 1/3rd of unconditioned microglial media. We assessed the effects of media conditioned by microglia for 12 or 36 h on this neuronotoxicity and measured the proportion of cells positive for markers of cell death at 5, 8, and 14 h of exposure to the media; at 5 and 8 h effects of media type on death were negligible (Supplementary Fig. 5).

**Fig. 5:**
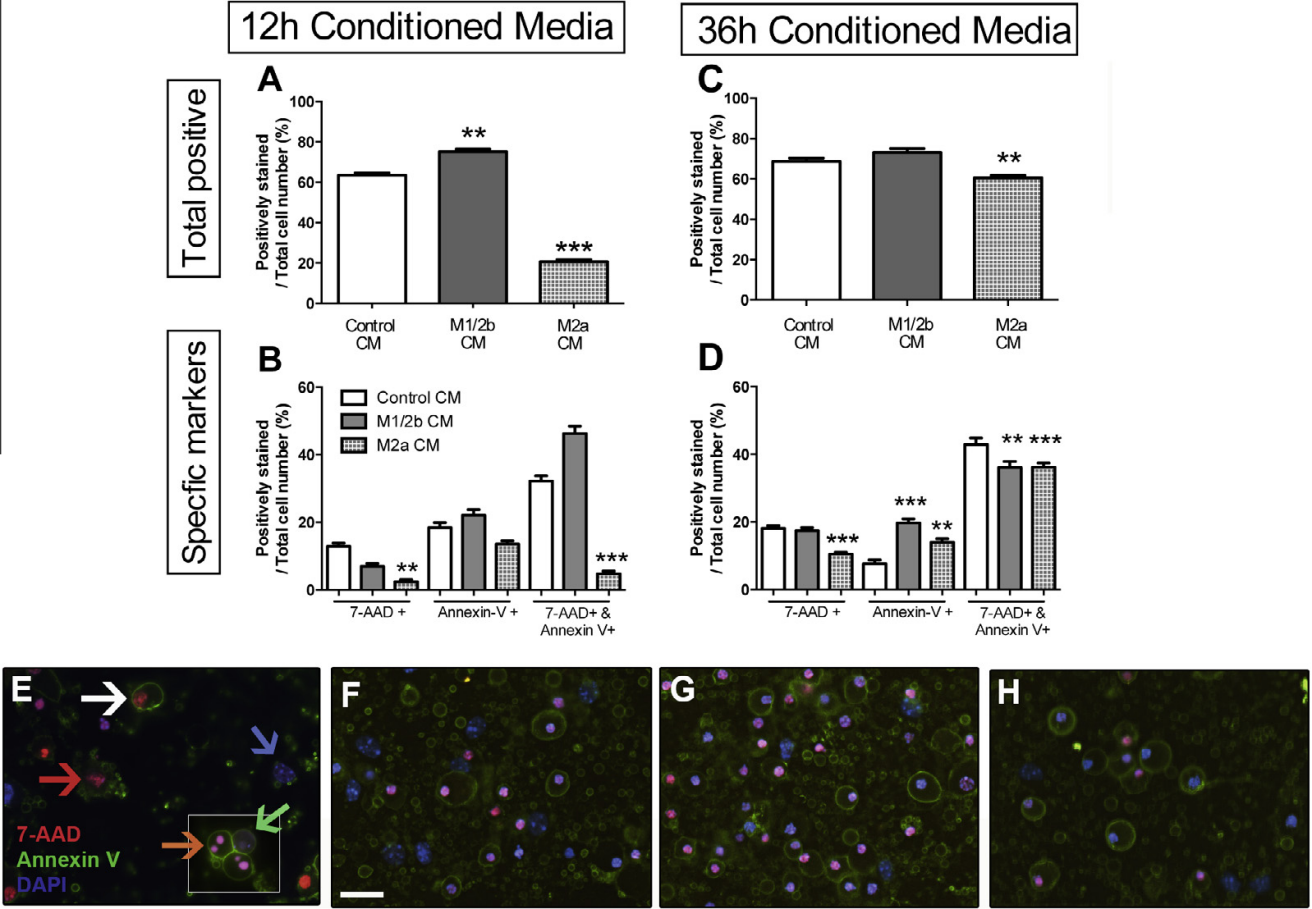
Phenotype of microglia alters the neuronotoxicity of conditioned media. Total appearance of cell death markers (A, C) and relative numbers of specific cell death type markers (B, D) in neurons exposed for 14 h to conditioned media harvested following 12 h (A, B, F–H) or 36 h (C, D) induction to an M1/2b or M2a phenotype, or PBS. Viability was assessed dependent on positive staining for 7-ADD, Annexin V, or both (see example, E), and specifically neurons were categorised as ‘normal’ (blue arrow) double negative and Hoechst positive, Annexin V positive only (red arrow), 7AAD+ positive only (green arrow), Annexin V+ and 7AAD+ with uncondensed nuclei (white arrow) or Annexin V+ and 7AAD+ condensed nuclei (orange arrow). Cells double positive with an uncondensed nuclei (white arrow) were very extremely rare. Representative images taken after exposure of neurons to 12 h CM for 14 h (F–H), illustrating basal staining of neurons in F (+PBS CM), increased cell death in +LPS CM (G), and deceased cell death in +IL-4 CM (H). Scale bar 25 μm. Data are mean ± SEM of at least 3 experiments. Data were assessed via an Kruskal Wallis, and where significant results of a Dunn’s post-test are shown; ^∗^*p* < 0.05; ^∗∗^*p* < 0.01; ^∗∗∗^*p* < 0.001, compared to PBS.

Factors in the media conditioned by M2a microglia for 12 h strongly reduced the total percentage of neurons positive for markers of cell death (Fig. 5A and B). More specifically (Fig. 5B), this media reduced the proportion of neurons positive for 7-AAD (necrotic-like) or Annexin V+/7-AAD+/condensed chromatin (late stage apoptotic-like). Where M2a microglia conditioned the media for 36 h, overall neuronotoxicity was reduced (Fig. 5C), but this effect was not as strong as for the media conditioned for 12 h.

In contrast, media conditioned by LPS treated microglia to become M1-cytotoxic caused a greater number of neurons to be positive for cell death markers after 14 h of growth factor deprivation. This is seen as a significant increase in the overall number of cells positive for cell death markers (Fig. 5A) or specific changes in the proportion of cells positive for Annexin-V+ (apoptotic-like death) (Fig. 5D).

### 3.6 Prior phenotype affects the subsequent expression of phenotype markers

**Fig. 6:**
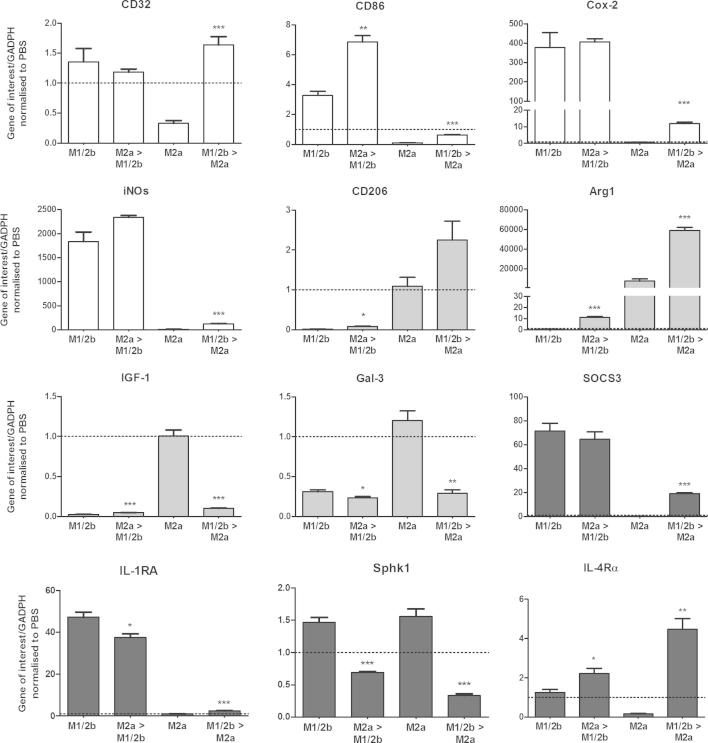
Prior phenotype alters the expression of phenotype markers in response to a second stimuli. Microglia were exposed to a single stimulus (LPS for 12 h or IL-4 for 36 h) or after one stimulus was removed it was replaced with the alternate stimulus as indicated. Gene expression is shown for markers grouped as M1 (white), M2a (grey) or M2b (black) dependent on their proposed function. Expression shown relative to PBS only treated control values from the respective time points, dotted line. Data are mean ± SEM of at least 3 independent experiments and assessed via Student’s *t*-test (primary stimuli vs. switched); ^∗^*p* < 0.05; ^∗∗^*p* < 0.01; ^∗∗∗^*p* < 0.001.

Inflammatory events influence the reactivity of glia in the brain long-term and any prior activation state of microglia is likely to alter the efficacy of immunomodulatory therapies (Nagamoto-Combs et al., 2007; Ramlackhansingh et al., 2011). In addition, microglia have been shown to be M2 immediately following injury so it is imperative to understand how they respond to further stimulation (Hu et al., 2012). As such, we characterised how prior phenotype alters development of the alternate phenotype. Microglia were induced to a maximal M1/2b phenotype (+LPS for 12 h), or a maximal M2a phenotype (+IL-4 for 36 h). The conditions were then switched, with LPS removed and replaced with IL-4 for 36 h or IL-4 removed and replaced with LPS for 12 h (Figs. 1 and 6).

Acquiring an M2a phenotype (+IL-4) before switching to an M1/2b (+LPS) caused expression of the M1 marker CD86 to be significantly greater (Fig. 6). However, previous exposure to an M2a inducing condition (+IL-4) before LPS did not prevent the typical induction of M1 markers (Cox-2, iNOS, CD32 and CD86) or the typical induction of M2b markers (IL-1RN, SOCS3 and IL-4Rα). Pre-acquisition of an M2a phenotype also prevented the typical reduction in the expression of IGF-1 caused by LPS (Fig. 6), but did not prevent LPS from reducing expression of the M2a markers Arg1, CD206 and Gal-3 (Mac-2).

Alternately, allowing microglia to develop into an M1/2b phenotype (+LPS) before switching to M2a inducing conditions (+IL-4) prevented the loss of M1 marker CD32 typically seen after IL-4 exposure. However, this did not prevent the typical reduction in M1 markers, Cox-2, iNOS, CD86 (Fig. 6).

Interestingly, acquisition of an M2a-repair/regeneration phenotype prior to induction of an M1/2b phenotype had contradictory and synergistic effect on SphK1 and IL-4Rα. SphK1 was reduced to below that observed in single exposure conditions alone and conversely IL-4Rα was greater compared to either single exposure conditions. Prior M1/2b phenotype had no effect on the characteristic decrease in the M2b markers, (SOCS3 or IL-1RA), in response to IL-4 (Fig. 6).

**Fig. 7:**
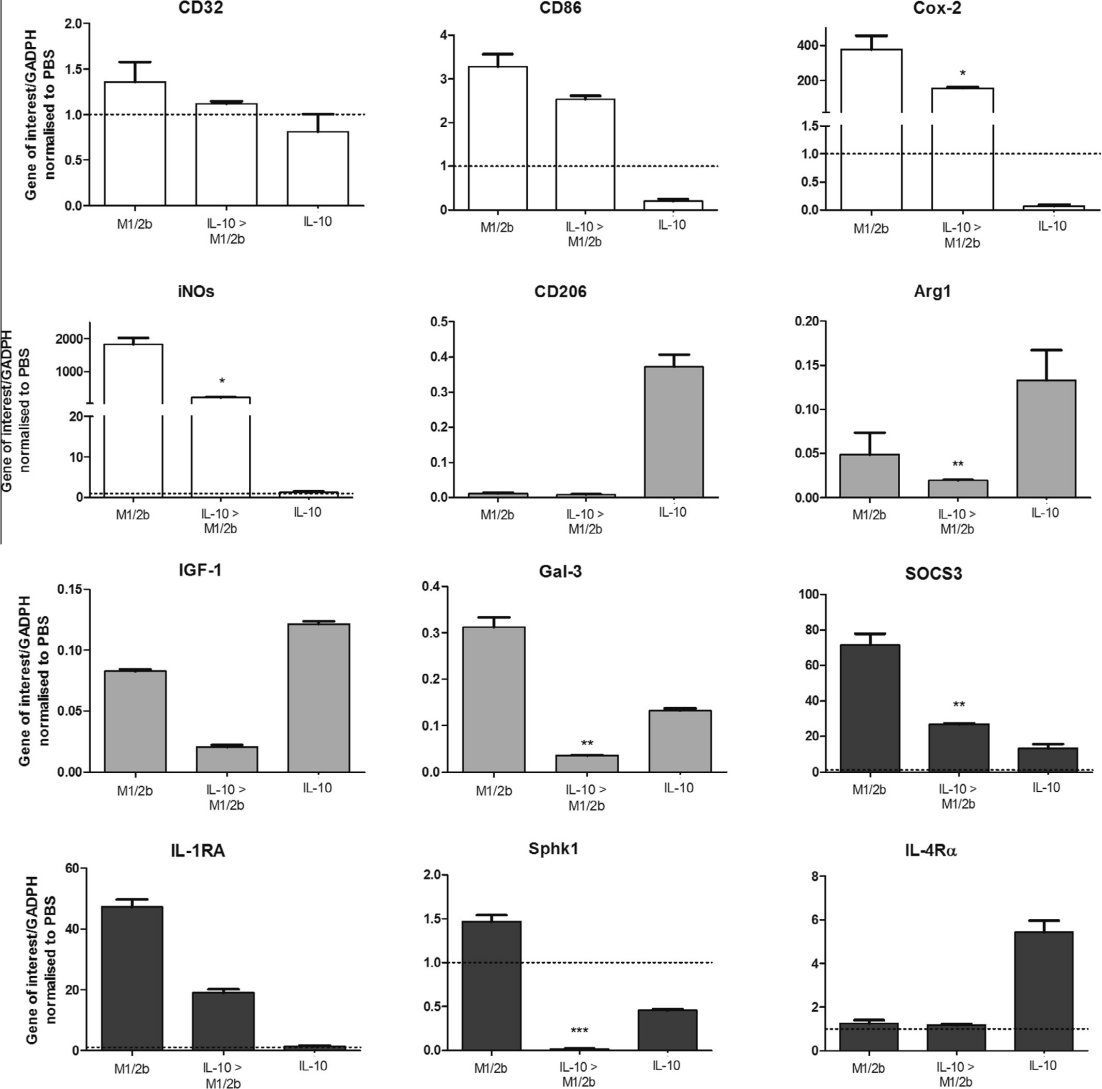
Prior IL-10 exposure impairs the development of a later M1/2b phenotype. Microglia were exposed to single or consecutive treatments of LPS (12 h) or IL-10 (12 h) as indicated and shown is gene expression for markers grouped as M1 (White), M2a (grey) or M2b (black) dependent on their proposed function. Expression shown relative to PBS only treated control values from the respective time points, dotted line. Data are mean ± SEM of at least 3 experiments assessed via Student’s t-test (primary stimuli vs. switched); ^∗^*p* < 0.05; ^∗∗^*p* < 0.01; ^∗∗∗^*p* < 0.001.

IL-10 induced a specific phenotype, and thus, we also assessed if acquisition of an M1/2b phenotype would alter the ability of IL-10 to induce our M2b markers (Fig. 7). Inducing an M2b only phenotype (+IL-10) before inducing an M1/2b phenotype (+LPS) prevented the typical response of increased iNOS, Cox-2, SphK1, and SOCS3. Moreover, pre-exposure to IL-10 exacerbated the typical reduction of Arg1 and Gal-3 by LPS, causing an even greater decrease (Fig. 7).

### 3.7 Prior phenotype reduces the release of neuronotoxic soluble factors

**Fig. 8:**
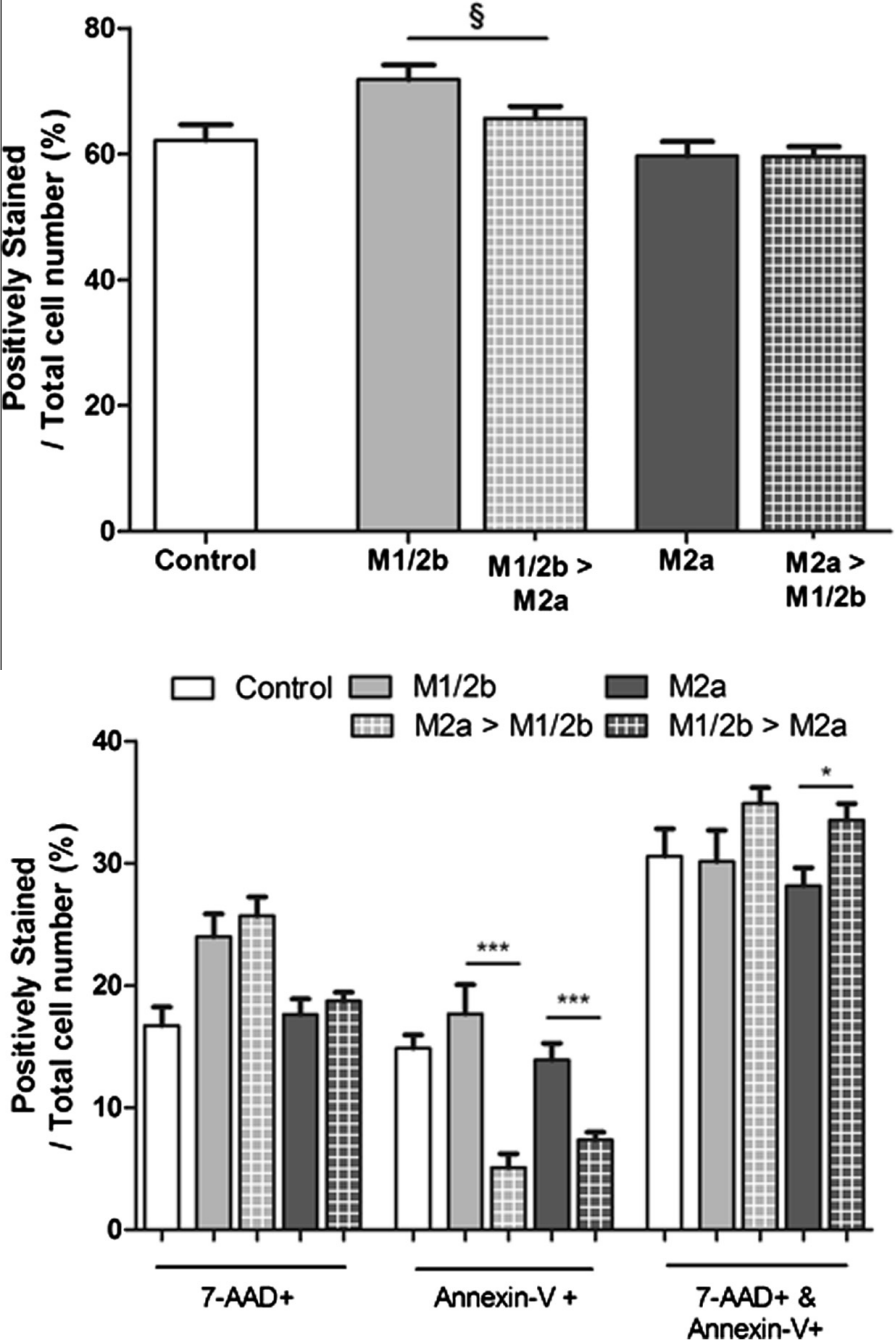
Prior phenotype reduces the neuronotoxicity of soluble factors from cytotoxic microglia. Neurons were exposed to conditioned media from microglia induced to a phenotype with a single stimulus (LPS 12 h or IL-4 36 h), or where the two treatments were applied in succession, with media changed between stimuli. After exposure to conditioned media for 14 h, the number of cells positive for cell death markers 7-AAD or Annexin V was assessed relative to total cell number (Hoechst positive). Data are mean ± SEM of at least 3 independent experiments assessed via Mann Whitney (single stimulus vs. switched); ^§^*p* = 0.05; ^∗^*p* < 0.05; ^∗∗^*p* < 0.01; ^∗∗∗^*p* < 0.001.

We must consider the effects of microglial products on neuronal survival following the combination of insult and immunomodulatory stimuli. As such, conditioned media was collected from the phenotype switching experiments and the effect of this prior phenotype on neuronal viability was assessed (Fig. 8). To do this we used the aforementioned growth factor deprivation model of neuronotoxicity and monitored the numbers of cells positive for Annexin V and/or 7-AAD, as surrogates for cell death. Prior induction of M2a phenotype (+IL-4) significantly reduced the toxicity of conditioned media from microglia then stimulated to become M1 cytotoxic with LPS (Fig. 8). Pre-induction to M1/2b phenotype (+LPS) did not alter the neuroprotective effects of media conditioned by subsequently IL-4 treated microglia. Interestingly, media from both switching paradigms caused less neurons to be positive for Annexin V, but this was greater for the IL-4-to-LPS media. In contrast, both switches appeared to increase the numbers of mixed Annexin V+ and 7-AAD+ cells, but this increase was only significant for IL-4-to-LPS conditioned media (Fig. 8).

## 4 Discussion

This aim of this study was to create a comprehensive analysis of microglial phenotype, including effects on neuronotoxicity, which can be used as a reference tool to identify the immunomodulatory potential of novel compounds. Inasmuch, for the first time this study outlines an *in vitro* model in which expression of thirty-one classical and more novel phenotype markers has been characterised in a time and stimulus dependent manner. All of the measured phenotype markers have important functions in inflammation. A complete review of their activities is outside the scope of this discussion, however, identifying how, and when, novel compounds modulate their expression is important in the design of viable efficacious neurotherapeutic paradigms. Functionally, we characterised how necrotic-like and apoptotic-like death is exacerbated by soluble factors from M1/2b induced microglia and reduced by soluble factors from M2a stimulated microglial. We also noted that prior phenotype had persistent and functional implications during the acquisition of a later phenotype.

### 4.1 Induction and timing of microglial phenotype

Our ability to identify the activation state of microglia is critical for timing any therapy. It was considered that an M1 cytotoxic phenotype develops due to insult/injury and that the same cell transitions to an M2-repair/regenerative phenotype over time (Stout et al., 2005; Kigerl et al., 2009; Perry et al., 2010). However, the presence of an early M2 phenotype (preceding M1) has recently been reported after hypoxic/ischemic injury *in vivo* (Hu et al., 2012). This study indicated that M2 markers were increased at 1 day, and persisted for up to 7 days after injury, overlapping with increases in M1 markers. In agreement with a prolonged evolution of a M2a repair and regeneration phenotype, key M2 markers were maximal following only 4 h of stimulation and soluble factors from this early response had the greatest neuroprotective effect. However, greater than half of the M2a markers did not peak in expression or release until far later (36–72 h). If there are discreet temporal phases of phenotype these may be druggable to affect neuroprotection. As such, assessment of temporal shifts in expression patterns may be as important as arbitrary increases or decreases in total activation when trialling therapies *in vitro*.

As just mentioned, soluble factors from the early phase (first 12 h) of M2a repair and regeneration microglia were more neuroprotective than products collected until maximal M2a gene and cytokine expression (at 36 h). Early increases in IGF-1 and TGFβ are likely important, given their known neuroprotective capacity (Henrich-Noack et al., 1994; Brywe et al., 2005). Also, although identification of novel soluble factors was outside the scope of this study, unknown microparticles (microvesicles, microRNA, bioactive lipids) (Antonucci et al., 2012), may have contributed to reduced neuronotoxicity and may themselves be interesting neurotherapeutics (Turola et al., 2012). Striking changes in the proportions of cells undergoing apoptotic or necrotic death were not observed. However, the mechanisms governing apoptosis and necrosis and as such the drugs targeting these processes are different. As such, when screening the effects of novel therapies including considering the utility of any pathway specific adjunct therapy, this analysis may be of importance.

### 4.2 Discriminating between microglial phenotypes

All pro-inflammatory stimuli induced a similar (if different magnitude) overall gene and cytokine response in microglia in general agreement with what has previously been reported (see, Colton, 2009; Ransohoff and Perry, 2009). Similarly, M1 markers can also be induced with beta-amyloid and agonists of group II metabotropic glutamate receptors and via other toll-like receptors in microglia and macrophages (Michelucci et al., 2009; Colton and Wilcock, 2010). This further indicates redundancy in this induction process, which has been suggested to involve the adaptor proteins MyD88 (Esen and Kielian, 2006; Dean et al., 2010).

Specifically, as previously reported for microglia and macrophage *in vivo* and *in vitro* LPS induced factors including iNOS, IL-1α, IL-6, CXCL1, CCL5 and TNFα (see, Colton, 2009; Ransohoff and Perry, 2009). Nitric oxide produced by iNOS is cytotoxic and each of these M1 markers is increased in brain and/or blood following brain injury or during inflammation (Dammann and Leviton, 1997; Hedtjarn et al., 2004; Helmy et al., 2011). In particular, increased IL-6 and TNFα is linked to a poor prognosis in infants suffering encephalopathy (Savman et al., 1998; Aly et al., 2006), these factors known to stimulate extrinsic pathways of cell death (Sidoti-de Fraisse et al., 1998). As such, these factors are assigned as M1-cytotoxic markers. We also observed that LPS increased expression of colony stimulating factors (G-CSF and GM-CSF), which may explain the increase in proliferation of microglia at 12 h (Giulian and Ingeman, 1988). Of note, the induction of an M1-cytotoxic phenotype also strongly decreased expression of M2a repair/regeneration markers, in agreement with reports that acquisition of phenotype requires a complex amalgam of induction and repression of gene expression (Liao et al., 2011).

Phenotypes have been considered separable stages in a temporally transitioning continuum (Mantovani et al., 2007; Kigerl et al., 2009). However, all pro-inflammatory stimuli induced M1 markers in parallel with the M2b markers SOCS3, SphK1 and IL-1RN. These 3 factors are associated with a M2b-deactivating or immunomodulatory phenotype, as SOCS3 reduces aberrant IL-6 family member activation (Croker et al., 2003); SphK1 catalyses the formation of sphingolipids with potent cell survival and proliferative effects (Bryan et al., 2008); and IL-1RA is an endogenous antagonist of IL-1 receptor activity (Akuzawa et al., 2008). We demonstrated that co-induction occurred as early as 2 h following stimulation, suggesting that these genes are independently induced. However, it is plausible that the rapid induction of Cox-2 by LPS drove increased M2b marker expression. Prostaglandins induce IL-1RN, SOCS3, SphK1/2, and IL-4Rα and as such, Cox-2 can be considered an M2b marker (Mosser and Edwards, 2008; Colton, 2009; Ransohoff and Perry, 2009).

Decreased expression of the T-cell stimulating factor IL-12 has been considered a hallmark of an M2b phenotype (Mantovani et al., 2009, David and Kroner, 2011). Although we did not observe this decrease, autocrine signalling and microglia-macrophage differences (discussed below) may explain this variation from previous reports.

Co-induction of cytotoxic and immunomodulatory markers, has previously been reported in the brains of adult mice following peripheral LPS (Fenn et al., 2012), and in macrophages *in vitro*, termed a hybrid phenotype (Mosser and Edwards, 2008). Increasing evidence indicates that diversity in marker expression is due to separable heterogeneous microglial populations, not simply co-expression of markers in the same cell (Lawson et al., 1990; Elkabes et al., 1996). Together with the temporal variation in marker expression, this governs the need to replicate both a wide selection and the time course to generate a first-line analysis of the effect of any therapeutic agent.

In response to IL-4, as previously reported, microglia increased the expression of CD206, Arg1, IGF-1, Gal-3, and CCR2 (Martinez-Pomares et al., 2003; Fenn et al., 2012). Expression of these markers is consistent with a repair/reparatory phenotype, as CD206 is a mannose receptor which stimulates phagocytosis (Zimmer et al., 2003); IGF-1 is a cytoprotective growth factor (Johnston et al., 1996); Arg1s catalytic activity produces polyamines that support extracellular matrix repair and mitochondrial function (Pesce et al., 2009); and Gal-3 is a modulator of proliferation (Inohara et al., 1998), and oligodendrocyte maturation and remyelination (Pasquini et al., 2011). Within this model system, increased Arg1 was the only marker able to positively discriminate an M2a phenotype at all time points. Bioavailability of the common precursor (arginine) of Arg1 and iNOS within the subcellular microdomains is thought to play an important role in phenotype, not the presence of the enzymes themselves (Hesse et al., 2001). However, we observed that microglia tended to predominantly increase expression of either enzyme suggesting, at least in this instance, it is enzyme levels that may play a role. The fibrous staining pattern we observed for Arg1 has been previously seen in LPS stimulated microglia (Scheffel et al., 2012), and may reflect increased transport via microtubules, reported previously in endothelial cells for arginase-2 (Ryoo et al., 2006). Exposure to IL-4 reduced the proliferation of microglia, unexpected as IL-4 has been previously shown to have no effect or to increase microglial proliferation *in vitro* (Suzumura et al., 1991, 1994).

### 4.3 A microglial molecular memory

Any immunomodulatory neurotherapy is going to interact with microglia already activated by insult or injury. It is therefore critical we understand how microglia respond when pre-conditioned. Furthermore, there is increasing clinical evidence of persisting microglial activation following brain injury, increasing susceptibility to neurodegenerative diseases. As such, we need to understand this molecular memory and any ability to switch the states of activated microglia (Long-Smith et al., 2009; Pinkston et al., 2009). Prior activation state synergistically and in a gene specific manner altered later development of an opposing phenotype. A similar complex synergy between phenotypes has been previously reported in microglia (Stout et al., 2005; Fenn et al., 2012). Of particular physiological relevance, prior exposure to IL-4 has been shown to inhibit the M1 response to beta-amyloid (Michelucci et al., 2009). Similarly, we observed that prior exposure to IL-4 altered the later response to LPS, and importantly, reduced the release of soluble neuronotoxic products from microglia. In macrophages, regions regulating transcriptional activity (enhancers) are modified by primary stimulus (such as LPS) leading to genes becoming promiscuous to secondary stimulus (such as IL-4) (Ghisletti et al., 2010). This phenomenon of cryptic enhancer region modification may underpin the molecular memory in this model. Further, we know that the activation of specific transcription factors is critical for the acquisition of phenotype (Stat1/6, PPARγ or Kruppel like factor) (Vats et al., 2006; Odegaard et al., 2007; Liao et al., 2011). However, little is known about the interactions between these factors and possible cross-regulatory signalling that might explain the modulation by switching, such as microRNAs (Jennewein et al., 2010).

### 4.4 Interleukin-10 an immunomodulatory stimulus

Exposure to IL-10 (or TGFβ or glucocorticoids) induces a specific phenotype in macrophages, termed M2c. This phenotype is characterised by high levels of anti-inflammatory cytokines, low levels of pro-inflammatory cytokines and increased IL-4Rα, Arg1, SOCS3 and CD206 (O’Farrell et al., 1998; Mantovani et al., 2004; David and Kroner, 2011). It is thus unsurprising that administration of IL-10 reduces experimental brain injury *in vivo* and *in vitro* (Spera et al., 1998; Molina-Holgado et al., 2001; Mesples et al., 2003). Interestingly, in our model IL-10 was the only stimuli to increase expression of IL-4Rα, in agreement with previous reports, but in contrast there was no loss of M2a-repair/regenerative marker expression (CD206 and Arg1) (O’Farrell et al., 1998; Imai et al., 2007). Also as expected, IL-10 prior to LPS inhibited M1-cytotoxic marker expression (O’Farrell et al., 1998; Kremlev and Palmer, 2005). Conversely, when the treatments were reversed (i.e. LPS before IL-10) expression of M2b-immunosuppresive markers was lower, indicating that microglia can lose the ability to respond to this anti-inflammatory stimulus.

### 4.5 Unique microglial activation

The origins and responsiveness to stimuli of microglia and blood-derived macrophages reportedly differs (Schmid et al., 2009; Dibaj et al., 2011; Saijo and Glass, 2011; Kierdorf et al, 2013). Differences in timing and dosages between studies limit any conclusive statements regarding microglia vs. macrophage responses from this data. However, in our study, compared to macrophages in response to IL-10 the Fcγ receptor CD16 was not increased (Wang et al., 2001), and IL-4 and LPS both reduced CX3CR1 (fractalkine receptor) expression (Ramos et al., 2010). These differences warrant further investigation to ensure that novel neurotherapeutics are optimised to modulate both the resident and infiltrating populations of immune cells participating in injury and repair processes.

In conclusion, we have presented a comprehensive set of data on phenotype markers and function that is unique in its inclusion of such a great number of classic and novel markers, and analysis over time. We also present additional support for the hypothesis of a functionally relevant microglial molecular memory that will be crucial in considering the *in vivo* response of microglia. Altogether, by using this data as a reference it will be possible to identify, as part of a first line screening process, the capacity for reducing cytotoxicity and/or supporting regeneration and repair of novel immunomodulatory compounds.

## Conflict of interest

All authors declare that there are no conflicts of interest.

## Author’s contribution

VC, TLC, MVO, JJ, and VD prepared the microglia and neuronal cultures. SL, VC, JJ, and VD performed the qRT-PCR and luminex assays. MVO, VC, TLC and EJ were involved with the neuronal death experiments. VC, ILC, KS, and CM were involved in the *in vitro* staining. BF and VC prepared the manuscript. VC, SL, EJ, HH, KS, CM, PG, and BF participated in experimental design and interpretation.

## Figures and Tables

**Table 1:** Markers assessed in this study whose expression in associated with specific phenotypes and their generalised functions; functions are known to overlap and be context dependent (Adapted and compiled from, Mosser and Edwards, 2008, Colton, 2009, Ransohoff and Perry, 2009, Mantovani et al., 2009).

	Proposed role in inflammation				
	Inflammotoxic	Pro-inflammatory	Anti-inflammatory	Repair	Immunoregulatory
M1 classic phenotype (cytotoxic)	TNFα	IL-1β			
	iNOS	IL-6			
		IL-12			
		IFNγ			
		CXCL1 (KC)			
		IL-1α			
		IL-2			
		CCL5 (RANTES)	Cox-2		

*M2a alternate phenotype* (*repair and regeneration*)			*IL-4*	*YM1*	*CD206 (MRC1)*
			*IL-1RA* (*IL-1Rn*)	*FIZZ1*	*Gal-3*
			*CX3CR1* (*fractalkine receptor*)	*IGF-1*	*CCL2* (*MCP-1*)
				*Arg1*	*CCR2* (*CD192*)
			TGF-β	*G-CSF*	
				*GM-CSF*	

M2b (c) Type II-deactivating phenotype (immunomodulatory)			IL-10		
			Cox-2		
			SphK1/2		
			SOCS3		
			IL-4Rα		

**Table 2:** Expression (gene, G or protein, P) after 12 h stimulation of phenotype markers relative to PBS only control. Data are mean ± SEM, for a minimum of *n* = 3 independent experiments.

Marker	Gene or Protein	Stimulus						
		LPS	IL-1β	TNFα	IL-4	IL-10	IFNγ	
CD16	G	0.43 ± 0.01	0.40 ± 0.01	0.36 ± 0.04	1.43 ± 0.06	0.62 ± 0.03	0.58 ± 0.03	M1, Cytotoxic
CD32	G	1.63 ± 0.26	1.04 ± 0.09	1.10 ± 0.22	2.04 ± 0.34	0.97 ± 0.23	0.03 ± 0.01	
CD86	G	2.33 ± 0.21	1.09 ± 0.12	1.03 ± 0.14	0.62 ± 0.10	0.15 ± 0.03	1.15 ± 0.17	
Cox-2	G	1248.00 ± 257.40	34.63 ± 4.06	45.70 ± 4.08	1.63 ± 0.28	0.25 ± 0.07	20.32 ± 9.82	
iNOS	G	2529.00 ± 279.80	215.10 ± 15.17	215.40 ± 16.05	1.06 ± 0.36	1.68 ± 0.33	85.38 ± 11.52	
IL-1β	P	283.60 ± 46.61	NA	206.20 ± 29.51	0.18 ± 0.07	2.77 ± 0.26	0.14 ± 0.03	
IL-6	P	18632 ± 4127.00	2396.00 ± 518.10	2999.00 ± 717.40	1.04 ± 0.48	20.01 ± 8.61	1.17 ± 0.15	
IL-12 (p70)	P	60.31 ± 0.86	15.89 ± 1.64	16.53 ± 0.77	9.22 ± 0.66	4.99 ± 0.91	0.48 ± 0.21	
TNFα	P	16085 ± 1931.00	315.90 ± 85.20	NA	1.02 ± 0.08	1.46 ± 0.51	1.07 ± 0.27	
CXCL1 (KC)	P	1518.00 ± 249.40	764.30 ± 128.90	1104.00 ± 158.00	0.96 ± 0.40	14.80 ± 1.38	0.73 ± 0.14	

CD206	G	0.01 ± 0.002	0.04 ± 0.003	0.04 ± 0.003	3.99 ± 0.45	0.47 ± 0.05	0.03 ± 0.001	M2a, alternative repair and regeneration
Arg1	G	0.48 ± 0.24	0.63 ± 0.30	0.50 ± 0.28	2763.00 ± 509.90	1.30 ± 0.33	0.22 ± 0.07	
IGF-1	G	0.08 ± 0.001	0.18 ± 0.02	0.18 ± 0.02	1.52 ± 0.08	0.12 ± 0.002	0.06 ± 0.001	
Gal-3	G	0.33 ± 0.02	0.36 ± 0.03	0.34 ± 0.02	2.03 ± 0.11	0.14 ± 0.01	0.72 ± 0.02	
CCR2	G	0.22 ± 0.02	0.17 ± 0.02	0.14 ± 0.003	1.13 ± 0.13	0.27 ± 0.05	0.70±0.07	
TGFβ	G	0.67 ± 0.01	0.95 ± 0.09	0.84 ± 0.05	1.03 ± 0.14	0.36 ± 0.07	0.56 ± 0.04	
CX3CR1	G	0.001 ± 0.0001	0.03 ± 0.01	0.02 ± 0.002	0.16 ± 0.02	0.14 ± 0.02	0.06 ± 0.01	
CCL2 (MCP-1)	P	2.22 ± 0.22	1.16 ± 0.21	1.35 ± 0.15	0.05 ± 0.003	0.23 ± 0.04	0.20 ± 0.01	

IL-1RA	G	109.80 ± 5.75	9.12 ± 0.95	8.21 ± 0.28	1.15 ± 0.17	3.12 ± 0.60	1.08 ± 0.08	M2b, immuno-modulatory
SOCS3	G	62.66 ± 5.66	10.11 ± 1.06	9.86 ± 1.09	0.66 ± 0.11	11.70 ± 2.11	3.81 ± 0.72	
Sphk1	G	2.54 ± 0.13	0.36 ± 0.12	0.39 ± 0.09	1.43 ± 0.14	0.79 ± 0.03	0.43 ± 0.04	
Sphk2	G	0.86 ± 0.05	0.69 ± 0.14	0.65 ± 0.07	0.59 ± 0.07	0.34 ± 0.06	0.53 ± 0.06	
IL-4Rα	G	1.15 ± 0.13	1.87 ± 0.20	1.49 ± 0.26	0.40 ± 0.05	4.97 ± 0.48	1.10 ± 0.13	
IL-10	P	61.52 ± 4.75	26.64 ± 2.23	23.59 ± 1.13	13.38 ± 0.41	NA	0.35 ± 0.10	

## Appendix A Supplementary data

Supplementary Table 1 Primer sequences, protein targets and NCBI references.

Supplementary Table 2 Expression (gene, G or protein, P) after 4 h stimulation of phenotype markers relative to PBS only control. Data are mean ± SEM, for a minimum of *n* = 3 independent experiments.
