# Supplementary material for: Characterization of phenotype markers and neuronotoxic potential of polarised primary microglia in vitro
Source: Brain Behav Immun. 2013 Aug;32:70–85. doi: 10.1016/j.bbi.2013.02.005 (PMC3694309; doi:10.1016/j.bbi.2013.02.005)
Supplement: Supplementary Table 1 — Primer sequences, protein targets and NCBI references. [file mmc1.doc]

**Supplemental Table 2: Expression (gene, G or protein, P) after 4 hours stimulation of phenotype markers relative to PBS only control. Data are mean ± SEM, for a minimum of n=3 independent experiments.**

| **Marker** | **Gene or Protein** | **Stimulus** | | | | | |  |
| --- | --- | --- | --- | --- | --- | --- | --- | --- |
|  |  | LPS | IL-1β | TNFα | IL-4 | IL-10 | IFNγ |  |
| CD16 | G | 0.38±0.05 | 0.63±0.10 | 0.50±0.03 | 2.01±0.10 | 1.48±0.25 | 3.60±0.47 | **M1, Cytotoxic** |
| CD32 | G | 1.87±0.19 | 2.54±0.39 | 2.16±0.39 | 3.07±0.13 | 3.61±0.48 | 1.26±0.29 |  |
| CD86 | G | 3.58±0.37 | 1.62±0.43 | 1.44±0.14 | 1.01±0.03 | 0.96±0.10 | 2.08±0.33 |  |
| Cox-2 | G | 292.80±23.79 | 27.14±6.51 | 32.66±7.42 | 2.85±1.79 | 4.49±0.24 | 9.72±4.64 |  |
| iNOS | G | 312.80±35.36 | 151.60±24.84 | 182.60±16.61 | 0.72±0.11 | 253.00±41.14 | 70.38±9.68 |  |
| IL-1β | P | 8.28±0.22 | NA | 3.59±0.46 | 2.52±0.15 | 2.51±0.18 | 0.99±0.19 |  |
| IL-6 | P | 130.70±6.35 | 12.22±1.63 | 8.35±1.72 | 0.86±0.47 | 1.24±0.43 | 0.51±0.29 |  |
| IL-12 (p70) | P | 5.86±0.29 | 3.12±0.16 | 2.81±0.35 | 3.67±0.23 | 2.28±0.08 | 0.75±0.15 |  |
| TNFα | P | 11206±495.20 | 299.90±89.55 | NA | 1.39±0.30 | 15.22±4.11 | 2.55±2.20 |  |
| CXCL1 (KC) | P | 25.37±2.89 | 10.39±4.22 | 9.61±2.66 | 0.69±0.42 | 1.11±0.55 | 0.49±0.30 |  |
| CD206 | G | 1.07±0.30 | 1.29±0.42 | 1.08±0.23 | 7.96±1.12 | 1.08±0.10 | 1.93±0.32 | **M2a, Alternative repair and regeneration** |
| Arg1 | G | 0.16±0.13 | 0.42±0.18 | 0.32±0.05 | 1150.00±264.40 | 1.53±1.15 | 0.24±0.15 |  |
| IGF-1 | G | 1.05±0.12 | 1.24±0.20 | 1.26±0.17 | 2.20±0.11 | 0.81±0.10 | 0.99±0.16 |  |
| Gal-3 | G | 0.69±0.03 | 0.81±0.08 | 0.74±0.02 | 1.79±0.38 | 0.62±0.004 | 0.98±0.16 |  |
| CCR2 | G | 0.24±0.03 | 0.23±0.05 | 0.32±0.07 | 0.84±0.18 | 0.43±0.08 | 1.50±0.19 |  |
| TGFβ | G | 1.40±0.14 | 1.23±0.08 | 1.33±0.13 | 1.37±0.05 | 1.01±0.16 | 0.91±0.11 |  |
| CX3CR1 | G | 0.28±0.05 | 0.32±0.06 | 0.36±0.08 | 0.59±0.16 | 0.35±0.09 | 0.17±0.05 |  |
| CCL2 (MCP-1) | P | 1.12±0.10 | 0.17±0.06 | 0.16±0.07 | 0.12±0.05 | 0.23±0.11 | 0.18±0.08 |  |
| IL-1Rn | G | 21.02±2.77 | 3.61±0.55 | 3.46±0.30 | 0.03±0.01 | 8.59±0.77 | 3.30±0.47 | **M2b, Immuno-modulatory** |
| SOCS3 | G | 141.90±20.91 | 31.91±2.08 | 41.78±5.09 | 2.14±0.84 | 83.28±20.66 | 14.80±4.47 |  |
| Sphk1 | G | 0.74±0.19 | 1.00±0.78 | 1.20±0.94 | 2.13±1.61 | 2.96±2.17 | 3.65±2.33 |  |
| Sphk2 | G | 0.97±0.12 | 0.81±0.03 | 0.93±0.10 | 1.60±0.23 | 1.46±0.41 | 0.83±0.17 |  |
| IL-4Rα | G | 1.44±0.13 | 1.10±0.40 | 0.99±0.16 | 2.28±0.15 | 10.50±3.06 | 4.68±0.18 |  |
| IL-10 | P | 15.99±1.10 | 5.29±0.69 | 4.19±0.56 | 15.19±0.30 | NA | 1.49±0.001 |  |
