# Supplementary material for: Characterization of phenotype markers and neuronotoxic potential of polarised primary microglia in vitro
Source: Brain Behav Immun. 2013 Aug;32:70–85. doi: 10.1016/j.bbi.2013.02.005 (PMC3694309; doi:10.1016/j.bbi.2013.02.005)
Supplement: Supplementary Table 2 — Expression (gene, G or protein, P) after 4 h stimulation of phenotype markers relative to PBS only control. Data are mean ± SEM, for a minimum of n = 3 independent experiments. [file mmc2.doc]

**Supplementary Table 1: Primer sequences, protein targets and NCBI references**

| **Gene** | **Target protein and abbreviation** | **Sense** | **Anti-sense** | **NCBI Reference** |
| --- | --- | --- | --- | --- |
| *GAPDH* | Glyceraldehyde 3 phosphate dehydrogenase (GAPDH) | GGC CTT CCG TGT TCC TAC | TGT CAT CAT ATC TGG CAG GTT | NM_008084.2 |
| *Inos* | Inducible nitric oxide synthase (iNOS) | CCC TTC AAT GGT TGG TAC ATG G | ACA TTG ATC TCC GTG ACA GCC | NM_010927.3 |
| *CD32* | Cluster of differentiation 32 (CD32) | CTG GAA GAA GCT GCC AAA AC | CCA ATG CCA AGG GAG ACT AA | NM_010187.2 |
| *CD86* | Cluster of differentiation 86 (CD86) | GAG CGG GAT AGT AAC GCT GA | GGC TCT CAC TGC CTT CAC TC | NM_019388.3 |
| *Ptgs2* | Cyclooxygenase-2 (Cox-2) | TCA TTC ACC AGA CAG ATT GCT | AAG CGT TTG CGG TAC TCA TT | NM_011198.3 |
| *CD16* | Cluster of differentiation 16 (CD16) | TAC ACA GCA CCA GTC CAA GC | AGA AAT AAA GGC CCG TGT CC | NM_010188.5 |
| *CD206* | Cluster of differentiation 206 (CD206) | CTT CGG GCC TTT GGA ATA AT | TAG AAG AGC CCT TGG GTT GA | NM_008625.2 |
| *Arg1* | Arginase-1 (Arg1) | GTG AAG AAC CCA CGG TCT GT | GCC AGA GAT GCT TCC AAC TG | NM_007482.3 |
| *Lgals3* | Galectin-3 (Gal-3) | GAT CAC AAT CAT GGG CAC AG | ATT GAA GCG GGG GTT AAA GT | NM_010705.3 |
| *Igf1* | Insulin like growth factor 1 (IGF-1) | TGG ATG CTC TTC AGT TCG TG | GCA ACA CTC ATC CAC AAT GC | NM_010512.4 |
| *Ccr2* | C-C chemokine receptor type 2 (CCR2) | GCC AGG ACA GTT ACC TTT GG | TTC CTG GTA GAG AGG CAA ACA | NM_009915.2 |
| *Tgfb* | Transforming growth factor β (TGFβ) | CTT TTG ACG TCA CTG GAG TTG | CAG TGA GCG CTG AAT CGA A | NM_011577.1 |
| *Cxc3cr1* | CX3C chemokine receptor 1 (CXC3CR1) | AAG TTC CCT TCC CAT CTG CT | GGA CAG GAA GAT GGT TCC AA | NM_009987.3 |
| *Sphk1* | Sphingosine kinase 1 (Sphk1) | TCC AGA AAC CCC TGT GTA GC | CAG CAG TGT GCA GTT GAT GA | NM_001172475.1 |
| *Sphk2* | Sphingosine kinase 2 (Sphk2) | CAG GGG TTA AGC CTG AGT GA | AGT CTG GCC GAT CAA GGA G | NM_001172561.1 |
| *Il1rn* | Interleukin 1 receptor antagonist (IL-1Rn) | TTG TGC CAA GTC TGG AGA TG | TTC TCA GAG CGG ATG AAG GT | NM_031167.5 |
| *Il4ra* | Interleukin 4 receptor alpha (IL-4Rα) | GGA TAA GCA GAC CCG AAG C | ACT CTG GAG AGA CTT GGT TGG | NM_001008700.3 |
| *Socs3* | Suppressor of cytokines 3 (SOCS3) | CGT TGA CAG TCT TCC GAC AA | TAT TCT GGG GGC GAG AAG AT | NM_007707.3 |
| *Retnla* | Found in inflammatory zone 1 (Fizz1) | AGG AAC TTC TTG CCA ATC CA | CAG TAG CAG TCA TCC CAG CA | NM_020509.3 |
